# Meeting Review: Bioinformatics and Medicine – From Molecules to Humans, Virtual and Real

**DOI:** 10.1002/cfg.178

**Published:** 2002-06

**Authors:** Roslin Russell

**Affiliations:** MRC UK HGMP Resource Centre Genome Campus Hinxton, Cambridge CB10 1SB UK

## Abstract

The Industrialization Workshop Series aims to promote and discuss integration, automation, simulation, quality, availability and standards in the high-throughput life sciences. The main issues addressed being the transformation of bioinformatics and bioinformaticsbased drug design into a robust discipline in industry, the government, research institutes and academia. The latest workshop emphasized the influence of the post-genomic era on medicine and healthcare with reference to advanced biological systems modeling and simulation, protein structure research, protein-protein interactions, metabolism and physiology. Speakers included Michael Ashburner, Kenneth Buetow, Francois Cambien, Cyrus Chothia, Jean Garnier, Francois Iris, Matthias Mann, Maya Natarajan, Peter Murray-Rust, Richard Mushlin, Barry Robson, David Rubin, Kosta Steliou, John Todd, Janet Thornton, Pim van der Eijk, Michael Vieth and Richard Ward.


**Comparative and Functional Genomics**
*Comp Funct Genom* 2002; **3**: 270–276.
Published online 9 May 2002 in Wiley InterScience (www.interscience.wiley.com). **DOI:** 10.1002/cfg.178

**Feature**

### Meeting Review: Bioinformatics And Medicine – From molecules to humans, virtual and real

Hinxton Hall Conference Centre, Genome Campus, Hinxton, Cambridge, UK – April 5th–7th

Roslin Russell*
*MRC UK HGMP Resource Centre, Genome Campus, Hinxton, Cambridge CB10 1SB, UK*

*Correspondence to: MRC UK HGMP Resource Centre, Genome Campus, Hinxton, Cambridge CB10 1SB, UK.

Received: 22 April 2002
Accepted: 24 April 2002

### Abstract

**The Industrialization Workshop Series aims to promote and discuss integration, automation, simulation, quality, availability and standards in the high-throughput life sciences. The main issues addressed being the transformation of bioinformatics and bioinformatics-based drug design into a robust discipline in industry, the government, research institutes and academia. The latest workshop emphasized the influence of the post-genomic era on medicine and healthcare with reference to advanced biological systems modeling and simulation, protein structure research, protein-protein interactions, metabolism and physiology. Speakers included Michael Ashburner, Kenneth Buetow, Francois Cambien, Cyrus Chothia, Jean Garnier, Francois Iris, Matthias Mann, Maya Natarajan, Peter Murray-Rust, Richard Mushlin, Barry Robson, David Rubin, Kosta Steliou, John Todd, Janet Thornton, Pim van der Eijk, Michael Vieth and Richard Ward. Copyright © 2002 John Wiley & Sons, Ltd.**

**Keywords:** **bioinformatics; medicine; virtual human; protein structure; personalized medicine; ontology**

### Introduction

The conference was jointly organized and sponsored by the Deep Computing Institute of IBM (**http://www.research.ibm.com/dci/**), the Task Force on Bioinformatics of the International Union of Pure and Applied Bioinformatics (**http://www.iupab.org/taskforces.html**) and the European Bioinformatics Institute (EMBL-EBI, **http://www.ebi.ac.uk/**). **Barry Robson** (**IBM Deep Computing Institute**) opened the workshop by stating that industrialization does not mean commercialization but high-throughput industrial strength. Last year saw the development of 'clinical bioinformatics' linking clinical data to genomics, requiring a large collective database that is 'patient-centric'. This field will lead to improved diagnostic tools, therapeutics and patient care.

### Keynote speakers

**François Cambien** (**Institut National de la Santé et de la Recherche Médicale, INSERM**) presented his latest results [9] and gave an overview on the molecular and epidemiological genetics of cardiovascular disease and the different approaches towards genotyping. He stressed the importance of creating a catalogue of 'all' common polymorphisms in the human genome. Since the start of the Etude Cas-Témoin sur l'Infarctus du Myocarde (ECTIM) in 1988 (the first large scale study on myocardial infarction, recent figures on the total number of candidate genes and polymorphisms across 29 studies are now at 102 and 475 respectively as recorded by GeneCanvas (**www.genecanvas.org**) a website supported by INSERM. The project and others will continue to search for more polymorphisms but

Copyright © 2002 John Wiley & Sons, Ltd.

how many are there, are they all relevant, and how do we make the most out of this information? Little is known about the distribution and structure of polymorphisms across the genome despite the availability of the almost complete sequence. On the assumption that there are 10–20 common polymorphisms per gene affecting coding and regulatory regions and that there are around 30 000 human genes in total, it is estimated that there may be around 500 000 functionally related SNPs. The classification and exploitation of polymorphisms for the whole genome not only requires data quality, storage, integration and accessibility, but also an understanding of data relevance and representation in relation to complex disorders. Lopez and Murray [5] ranked cardiovascular disease the fifth most common disease in 1990 but they estimate that in 2020, it will be the most common disease worldwide. There is a complex interplay between environmental and genetic determinants: the former may explain the difference in disease prevalence between population groups, and time related changes, while the latter plays a role in susceptibility. However, the expectations of finding genetic factors for multifactorial diseases may not be fulfilled because many genes are involved and several models (such as the whole genome approach) may be much too simplistic. Important correlations may also be found not only in phenotypes such as clinical data but also in biological systems. When integrated with epidemiology and Mendelian genetics, the application of high throughput proteomic tools as well as comparative investigations of different species will provide a greater understanding into evolution and functional biology and is expected to provide new leads for drug development or other approaches to maintain health.

**Janet Thornton** (**EMBL-EBI**, **http://www.ebi.ac.uk/Thornton/index.html**) presented results on the analysis of the domain structure of enzymes and how this knowledge can be interpreted in the context of the evolution of metabolic pathways [10,8,6]. The results from an analysis of small molecule metabolic pathways in yeast support a ‘mosaic’ model of evolution in which enzymes in metabolic pathways are chosen in an unsystematic way to meet the functional requirements of a metabolic pathway. Further analysis on the conservation of function across families of homologous enzymes shows that the degree of functional similarity varies according to the degree of sequence identity shared by enzyme homologue pairs. High sequence identity corresponds to almost identical function, while proteins with less than 30% sequence identity show significant functional differences. These results have been applied to the problem of genome annotation and functional assignments have been catalogued in the database, Gene3D (**http://www.biochem.ucl.ac.uk/bsm/cath_new/Gene3D/**) for a number of completed genomes, providing functional and structural annotation.

### Virtual human biology

**Michael Ashburner** (**University of Cambridge**) spoke about recent developments and tools associated with the Gene Ontology™ Consortium (GO, **http://www.geneontology.org/**). GO uses a natural language ontology system and aims to provide a set of structured vocabularies for specific biological domains that can be used to describe gene products in any organism. There are GO terms for human, mouse, rat, worm, fly, amoeba, yeast, *Arabidopsis* and the more recently sequenced rice, and TIGR are now remodeling all their bacterial genomes. The three organizing principles (orthogonal ontologies) of GO are: biological process, molecular function and cellular component. There is a hierarchical structure within these groups that allows querying at different levels and as the number of these parent-child relationships increase, the annotations get more complex. Many databases are now collaborating with GO and these include MGI, LOCUSLINK, SWISSPROT and CGAP. Recent browsers and query tools attached to GO include the following: the MGI GO Browser, AmiGO (Berkeley, **http://www.godatabase.org/**) ; the CGAP GO Browser (**http://cgap.nci.nih.gov/Genes/GOBrowser**), the EP : GO Browser (**http://ep.ebi.ac.uk/EP/GO/**) which is built into EBI’s Expression Profiler (a set of tools for clustering and analysing microarray data); QuickGO (**http://www.ebi.ac.uk/ego/**) which is integrated into InterPro; and DAG-Edit (**http://sourceforge.net/projects/geneontology**), a purpose built, downloadable Java application to maintain, edit and display GO terms. With the emerging development of ontologies in other domains, the Global Open Biology Ontologies (GoBo) or Extended GO (EGO) has also been set up with the aim of being open source and DAG-Edit compatible, using non-overlapping defined terms and common ID space.

**Richard Ward** (**Oak Ridge National Laboratory**)

Copyright © 2002 John Wiley & Sons, Ltd. 

is involved in the Virtual Human (VH) Project (**http://www.ornl.gov/virtualhuman/**), a human simulation tool. The ambitious goal of the VH will be an integrated digital representation of the functioning human from the molecular level to the living human, and from conception to old age. Tools are being developed to represent the body's processes from DNA molecules and proteins to cells, tissues, organs and gross anatomy. These tools include XML (Extensible Markup Language) where ontologies and a high level description of the human body are important, visualization tools and problem solving environments. A forum has been established for the digital human effort to provide open source code tools to represent the body's processes: **http://www.fas.org/dh/**. The Physiome project (**http://www.physiome.org**) is another comprehensive modeling environment that aims to promote modeling for analysing integrative physiological function in terms of underlying structure and molecular mechanisms. There is a recognised need to adopt standards for data definition, storage and transfer, XML to replace standard data input, and databases for creating and developing XML schemas. There is also a need to establish integrative databases dealing with genomic, biophysiological, bioengineering and clinical data. XML descriptions are now becoming more common and examples of these include: cellular function, cellMEL (**http://www.cellml.org**); systems biology modeling, SBML (**http://xml.coverpages.org/sbml.html**); and their own prototype for physiological modeling, physioML (**http://www.ornl.gov/virtualhuman**). His group originally used a Java SAX parser for physioML but they converted to using a Java document object model instead, since new objects in the description are easily defined and it allows the integration of physiology and anatomy. However, ontologies have an advantage over XML because they provide a richer description of relationships (semantic rules) between objects and interoperability improves between systems. They are now creating a Digital Human (DH) for the development of an anatomical ontology. The DH requires a problem-solving environment for complex 3D-flow modeling and for high performance computing, three-tiered client/server architecture with Java RMI and NetSolve (**http://icl.cs.utk.edu/netsolve**) is used. He also mentioned how a common component architecture, which is currently used for climate modeling, is the way of the future for the VH project.

### Protein structure and protein interaction

**Jean Garnier** (**INRA – de Recherche de Versailles**) gave an overview of protein structure prediction techniques, describing some of the more widely used tools. Structure prediction tools fall into three classes, those which predict structures from first principles such as molecular dynamics simulations, those based on statistical analysis, such as hidden Markov models (HMMs) and those based on comparison to known structures, i.e. homology modeling. Jean Garnier stressed the importance of testing different techniques, referring to EVA and CASP. EVA is an automatic evaluation tool that continuously tests publicly available structure prediction servers by feeding them sequences for known new structures from PDB and comparing the results with the determined structures. CASP is a public competition in which experts attempt to predict structures for proteins whose structures have been determined but whose structures have not been made public. So far, structure prediction is most successful for proteins with known folds. Identification of proteins whose structure may be similar to a novel protein is often difficult and attempts to identify homologues of a new sequence often fail despite the existence of homologues with known structures because linear alignment of sequences is not as conserved as a three-dimensional topology. Some recent 'threading' programs have been developed with better sensitivity than conventional alignment techniques for detecting distant homologues for novel sequences, such as FORESST (**http://abs.cit.nih.gov/foresst/**, 3) and FROST (**http://www.inra.fr/bia/produits/logiciels/BD2html.php?nom=FROST**). The former program predicts which known fold best matches a novel sequence using HMMs based on patterns of secondary structure in proteins with known structures to analyse novel proteins. Secondary structure prediction algorithms are applied to the novel sequence to determine a predicted secondary structure topology which is then fed to the HMM to identify known structures with matching topologies.

Major evolutionary transitions have arisen as a result of changes in protein repertoires and changes in the expression of protein repertoires. **Cyrus Chothia and Bernard de Bono** (at the **MRC Laboratory of Molecular Biology**) have been studying the immunoglobulin superfamily as a model of whole genome protein repertoires. In his talk Dr

Copyright © 2002 John Wiley & Sons, Ltd. 

Chothia discussed problems that have arisen from analyses of Ensembl in the context of attempts to determine the complete repertoire of immunoglobulin superfamily proteins in the human genome. Application of HMMs to predicted proteins from Ensembl to identify immunoglobulin superfamily proteins is hampered by various issues. Parts of some genes are absent while parts of other genes are split and widely separated from each other. Consistency between releases of Ensembl is also a problem. Each new Ensembl release contains new protein predictions, while predictions from previous releases may be deleted. The solution involved looking at all nine releases of Ensembl to collectively identify all immunoglobulin superfamily proteins from all the predicted proteins in these releases using HMMs. This was followed by removing redundant predictions and confirming the quality of the predictions that remain using GeneWise.

### Environmental healthcare issues and bioinformatics/individuality and the pursuit of personalised molecular medicine

**Kenneth Buetow** (**NCBI**) is actively involved in the Cancer Genome Anatomy Project (CGAP) and spoke about how combining bioinformatics and genomics gives an insight into the etiology of cancer. The most obvious challenges associated with utilizing large data collections are management, visualisation and interpretation. If bioinformatics is to succeed in integrating knowledge from diverse fields of research, the problem of standardizing terminology and language must be overcome. He discussed how the NCBI is using the GO™ infrastructure in cancer research.

The annotation of genomes requires sensitive tools for the identification of homologous proteins, allowing proteins of unknown function to be linked to homologues with known function. Conventional sequence alignment tools are often insensitive to distant sequence relationships even though the distantly related sequences may have similar structures and functions. Since structure is typically more conserved than sequence, the use of structure based homologue recognition tools for genome annotation has significant value. **Michael Sternberg** (**Imperial College**) presented the latest results from his group on the development of genome annotation tools based on structural profiles [2,7]. His group has developed tools both to identify distantly related homologues and to assign function to those homologues. The 3D-PSSM tool (**http://www.sbg.bio.ic.ac.uk/~3dpssm/**) was developed to improve PSI-BLAST predictions and to detect distant sequence relationships using structural profiles from known proteins. An algorithm has also been developed for predicting functional sites in proteins based on multiple sequence alignment of homologous sequences followed by identification of invariant polar residues. Spatially clustered groups of these residues are considered to be functional sites. Comparison of data for proteins with known functional sites with the multiple alignment predictions showed the system to be 94% accurate. He emphasized the extent to which automated methods can be used but also how human insight can provide added value to genome annotation projects.

It is proving difficult to develop novel therapeutic molecules for treatment of multifactorial disorders based upon known targets despite a steady increase in the number of such targets. In most cases, therapeutic molecules, which successfully alleviate a disease do not directly affect the causative gene products, instead they act on the physiological mechanisms that are ultimately triggered. Therefore, there is a need to identify these physiological mechanisms and an understanding of the underlying events should be the main aim of the research. However, technological and conceptual difficulties must be resolved for functionally targeted drug development to be a reality. Since it is well established that physiological changes result from and induce gene expression pattern changes, the latter together with pathological states could be used to determine the physiological mechanisms implicated. However, it is important to consider the isoforms and any protein modifications involved, the types of complexes they associate with and the nature of the systemic environment. **François Iris** (**Hemispherx Biopharma Europe**) presented Bio-Graph, a computer-assisted approach to the determination of phenotype-specific physiological mechanisms for drug discovery. He pointed out the data mining problems of 'textual informatics' and discussed how to approach them. Biograph is an integrative systematic approach that is a four-phased physiological modeling process. It takes information from diverse forms and formats to create pertinent knowledge that is directly implemented to tackle the biological problems being addressed. In the first phase, data from various biological databases (for

Copyright © 2002 John Wiley & Sons, Ltd.

examples, MedLine, OMIM, KEGG and SPAD) are mapped into a relational graph, and then in the second phase, the graph is interpreted in the context of the problem of interest, leading to a sub-graph that reveals potential pathological links. The third phase involves the construction of a theoretical physiological model entirely supported by scientific literature and finally, the last phase identifies pathway-associated potential intervention points and therapeutic intervention modes. Direct experimental biological verification of the results is then required.

**Kosta Steliou** (**Boston University**) gave an overview of population history and genomics. Studies into patient history and genealogy give important insights into the molecular basis of disease. He described how the analysis of the mitochondrial genome can be used to segregate patients into medically relevant genetic subgroups. Research has shown that there are certain mutations in the mitochondrial DNA that are associated with diverse diseases such as cancer, infertility, diabetes, stroke, deafness and many others (**http://www.mitoresearch.org**). There are also associations between the A3243G mtDNA mutation and neurodegenerative disorders.

### Researching and simulating biomolecular interaction networks and bioinformatics aspects of intercellular integration

Despite the ease with which SNPs can be identified, determining the biological significance of SNPs provides many challenges. In his presentation, **John Todd** (**University of Cambridge**) gave examples from his work in type I diabetes (TID), and autoimmune disease. He pointed out that there are two general mechanisms by which SNPs have their effect, either by changing the regulation of the gene or by changing the precise amino acid sequence coded by the gene. Analysis of the function of SNPs is complicated by epistasis (which is where the effects of one SNP may be masked by another). To deal with epistasis, large sample sizes are required and Todd suggested that a case study of around 5000 samples is required. This implies that SNP screening must become an industrial process once SNPs are recognized in all haplotypes. However, the technology is still approximately 12-fold out in terms of cost therefore more cost effective methods are required. Genetics has to scale up to enable population-based studies and he currently has funding for a case study of 10 000 DNA patient samples from the UK Paediatric TID registry. The 'registries' provide a diverse range of cases, patient history and other relevant clinical data such as reasons for death. Another challenge in SNP research is in understanding splicing complexity.

**Matthias Mann** (**University of Southern Denmark and MDS-Proteomics**) presented the latest results from his collaborative work on high-throughput mass spectrometric protein complex identification [4]. Libraries of expressed cDNAs encoding 'bait' proteins are prepared by a high-throughput recombination procedure such that the members of the library of expressed proteins are each linked to an epitope tag. This tag may be used to immunoprecipitate the expressed 'bait' protein and any proteins that are interacting with the 'bait' protein from whole cell extracts. The components of the immunoprecipitated complex can then be identified further by gel electrophoresis and peptide mass fingerprinting. The technique found 3-times more interactions than the yeast 2-hybrid technology in a comprehensive study of the yeast proteome. Some teething troubles were mentioned such as ectopic expression of the tagged clones and a significant number of false positives. The filtered data set has been deposited in BIND™, a powerful protein-protein interaction database (**http://www.bind.ca/**). Raw data can be obtained from **http://www.mdsp.com/yeast**.

Matthias Mann also referred to his latest work in collaboration with Angus Lamond in Dundee on the characterisation of the nucleolus, a substructure within the cell nucleus [1]. This project aims to determine which proteins are found in the nucleolus, what they interact with, and what their function is. Nucleolar isolation followed by 2-D gel electrophoresis and peptide mass fingerprinting has been used to identify components of the nucleolus. Issues that have arisen from this project include how to confirm that proteins identified by this process actually reside in the nucleolus rather than being contaminants. Fluorescence localization using Yellow Fluorescent Protein tagged clones was carried out to confirm nucleolar residence of the proteins identified by mass spectrometry.

Matthias Mann emphasized the need for standards to interpret proteomic data particularly given the variety of analytical methods that are referred to as proteomic techniques. Predictive tools may become more valuable for functional pathways once more data is available, such as more *in vivo* data on

Copyright © 2002 John Wiley & Sons, Ltd.

phosphorylation sites to allow the identification of unknown proteins with phosphorylation sites.

**David Rubin** presented Cognia, a company that is developing proteomic database products. Cognia has licensed the right to distribute commercial versions of the TRANSFAC and TRANSPATH databases with improved querying tools and interfaces. Cognia is also developing a proprietary relational database product, the Protein Catabolism DataBase (PCDB), a comprehensive database on protein turnover and protein degradation pathways. This will be of value in a number of diseases in which protein turnover is believed to be aberrant, such as in certain cancers, neurodegenerative diseases and inflammatory disorders. The PCDB database includes data on degradation mechanisms, post-translational modifications, structure, protein-protein interactions and signal transduction. The PCDB product includes search tools to enable Boolean queries, structural searching and tools to enable tailored curation specific to particular organizations or projects. The design of the database is amenable to inclusion of open source code.

Molecular docking is a technique for determining how well pairs of molecules interact with each other. Pairs of molecules are 'docked' in various orientations and the energy of each interaction is scored. The process is repeated to find the best interaction between the pair. Applications include '*in silico*' drug screening to filter compound libraries prior to actual screening and prediction of binding conformation of hits from screening. **Michael Vieth** (**Eli Lilly and Company**) addressed various limitations of docking algorithms. The flexibility of proteins, and their ability to adopt novel conformations on ligand binding, are difficult to account for, as are changes in ligand conformation. Multiple X-ray structures and improved Structure Activity Relationships (SARs) from known proteins/ligand complexes can improve predictions for new proteins and/or new ligands.

### E-Medicine, medical research and healthcare in an Internet world

**Maya Natarajan** (**Entropia, Inc.**, **http://www.entropia.com/**) presented results of how 'grid computing' aka 'distributed computing' can accelerate bioinformatics research. Using BLAST results and results from docking experiments from a pharmaceutical company, she demonstrated how moderately sized networks of PCs that are actively performing menial tasks can provide significant computational power for processor intensive problems. The computational power derived from such networks grows linearly with the number of computing nodes within the network and the use of such networks provides a cost-effective solution for high performance computational needs.

**Barry Robson**'s presentation on 'Health Care and Personalised Medicine' was a visionary account of what the healthcare system might be like in the future and how this may be achieved. The medicine of the future will take advantage of personal genomic and expression data but such data cannot be used to its full potential unless there is an effective link and accessibility to patient records. IBM's vision for a global healthcare system, 'The Secure Health and Medical Access Network' (SHAMAN) provides the infrastructure for this goal. The future prospect of designing personal drugs in real time is achievable. The drugs will be designed for patients on high performance servers at pharmaceutical companies and FDA offices. However, such a vision will mean that a change in the healthcare system is required. The development of SHAMAN essentially requires the encoding of patient record into digital form and the team is working closely with a group in Oxford on bioethics issues. An XML standard is used for the 'clinical' document 'architecture' and various forms of visualization tools are being developed. Mitochondrial DNA may be used as a 'barcode' for the record. A major problem is that the data is dirty, sparse and in a high dimensional space. Algorithms are required to cope with this, for example, knowledge can be reduced into a few terms using mathematical terminologies. Extensions to SHAMAN will include a database of potential pathogens and also a database of animal models for human disease.

**Richard Mushlin** (**IBM T. J. Watson Research Center**) mentioned that digital patient data has been around for many years now, and the recent success of these systems is largely due to the diverse forms of data being structured according to standards and brought under the control of integrated applications. Several of these applications are complete enough such that virtually no paper work is involved. However, with the emergence of new multi-source data types such as personal genomic information, these systems must be able to accommodate them electronically, or risk returning to paper records. He emphasized the importance of integrating genomic

Copyright © 2002 John Wiley & Sons, Ltd. 

data with clinical data for making scientific discoveries. Re-evaluation tools for the data will be required as more information is fed to the system.

**Pim van der Eijk** (**Oasis**, **http://www.oasis-open.org/**) gave a high-level introduction to XML and presented the results of a survey of several XML-based languages in biology with the emphasis on schema design, process issues and XML-based knowledge formalisms.

**Peter Murray-Rust** (**Unilever Centre for Molecular Informatics**) described an XML based system for chemical nomenclature. He emphasized the need for agreed ontologies and discussed the problems of overlapping domains. Scientific publication can be used to create a knowledge base, or semantic web, and this has great potential in providing novel knowledge discoveries beyond what is presently found in traditional publications.

### Conclusion

A diverse array of subjects was covered by the speakers and a number of underlying themes have emerged relating to the exploitation of genomic data, principally the need for standardization both of data structure and vocabulary. To achieve this goal of integration of disciplines as varied as clinical genetics, structural genomics and proteomics, this process of standardization will need to be far-reaching and extensive, and achieve global acceptance. Scientific publication can also provide and create a wealth of knowledge, however, publication will need to be in an accessible format for data integration and mining. Furthermore, if medical research is to evolve, academics in particular need to take an 'industrial approach' to research in order to achieve the necessary scale of data acquisition and management for genome-wide population based studies. To successfully scale up, research processes will require effective automation particularly of genome annotation and the task of assigning functions to novel sequences. High throughput structure analysis is beginning to provide automated methods of genome annotation. Accurate functional annotation of genomes will lead to better interpretation of both molecular and clinical genetics, allowing structural and functional information to be linked to clinical phenotypes. The ultimate outcome of this process of integration will be improved health-care for all.

### References

1. Andersen JS, Lyon CE, Fox AH, *et al.* 2002. Directed proteomic analysis of the human nucleolus. *Curr Biol* **12**: 1–11.
2. Bates PA, Kelley LA, MacCallum RM, Sternberg MJ. 2001. Enhancement of protein modeling by human intervention in applying the automatic programs 3D-JIGSAW and 3D-PSSM. *Proteins* **45**: 39–46.
3. Di Francesco V, Munson PJ, Garnier J. 1999. FORESST: fold recognition from secondary structure predictions of proteins. *Bioinformatics* **15**: 131–140.
4. Ho Y, Gruhler A, Heilbut A, *et al.* 2002. Systematic identification of protein complexes in *Saccharomyces cerevisiae* by mass spectrometry. *Nature* **415**: 180–183.
5. Lopez AD, Murray CC. 1998. The global burden of disease, 1990–2020. *Nat Med* **4**: 1241–1243.
6. Rison S, Teichmann SA, Thornton JM. 2002. Homology, pathway distance and chromosomal localisation of the small molecule metabolism enzymes in *Escherichia coli*. *J Mol Biol* **00**: 00–00.
7. Smith GR, Sternberg MJ. 2002. Prediction of protein-protein interactions by docking methods. *Curr Opin Struct Biol* **12**: 28–35.
8. Teichmann SA, Rison SC, Thornton JM, Riley M, Gough J, Chothia C. 2001. The evolution and structural anatomy of the small molecule metabolic pathways in *Escherichia coli*. *J Mol Biol* **311**: 693–708.
9. Tiret L, Poirier O, Nicaud V, *et al.* 2002. Heterogeneity of linkage disequilibrium in human genes has implications for association studies of common diseases. *Hum Mol Genet* **11**: 419–429.
10. Todd AE, Orengo CA, Thornton JM. 2001. Evolution of function in protein superfamilies, from a structural perspective. *J Mol Biol* **307**: 1113–1143.

Copyright © 2002 John Wiley & Sons, Ltd. 

## References

[PDF_00636] Andersen Jens S., Lyon Carol E., Fox Archa H., Leung Anthony K. L., Lam Yun Wah, Steen Hanno, Mann Matthias, Lamond Angus I. (2002). Directed proteomic analysis of the human nucleolus.. Curr Biol.

[PDF_00639] Bates P. A., Kelley L. A., MacCallum R. M., Sternberg M. J. (2001). Enhancement of protein modeling by human intervention in applying the automatic programs 3D-JIGSAW and 3D-PSSM.. Proteins.

[PDF_00643] Di Francesco V., Munson P. J., Garnier J. (1999). FORESST: fold recognition from secondary structure predictions of proteins.. Bioinformatics.

[PDF_00646] Ho Yuen, Gruhler Albrecht, Heilbut Adrian, Bader Gary D., Moore Lynda, Adams Sally-Lin, Millar Anna, Taylor Paul, Bennett Keiryn, Boutilier Kelly (2002). Systematic identification of protein complexes in Saccharomyces cerevisiae by mass spectrometry.. Nature.

[PDF_00649] Lopez A. D., Murray C. C. (1998). The global burden of disease, 1990-2020.. Nat Med.

[PDF_00655] Smith Graham R., Sternberg Michael J. E. (2002). Prediction of protein-protein interactions by docking methods.. Curr Opin Struct Biol.

[PDF_00659] Teichmann S. A., Rison S. C., Thornton J. M., Riley M., Gough J., Chothia C. (2001). The evolution and structural anatomy of the small molecule metabolic pathways in Escherichia coli.. J Mol Biol.

[PDF_00662] Tiret Laurence, Poirier Odette, Nicaud Viviane, Barbaux Sandrine, Herrmann Stefan-Martin, Perret Claire, Raoux Ségolène, Francomme Carole, Lebard Géraud, Trégouët David (2002). Heterogeneity of linkage disequilibrium in human genes has implications for association studies of common diseases.. Hum Mol Genet.

[PDF_00666] Todd A. E., Orengo C. A., Thornton J. M. (2001). Evolution of function in protein superfamilies, from a structural perspective.. J Mol Biol.

